# Vitamin K deficiency bleeding in an apparently healthy newborn infant: the compelling need for evidence-based recommendation

**DOI:** 10.1186/s13052-019-0625-y

**Published:** 2019-03-04

**Authors:** Simone Ceratto, Francesco Savino

**Affiliations:** 1 Department of Public Health and Pediatric Sciences, Postgraduate School of Pediatrics, University of Turin, Turin, Italy; 2 Early infancy Sub-intensive Care Unit, Città della Salute e della Scienza di Torino, “Regina Margherita” Children’s Hospital, Piazza Polonia, 94, 10126 Turin, Italy

**Keywords:** Newborn infants, Vitamin K prophylaxis, Intracranial bleeding

## Abstract

**Background:**

Vitamin K is a key point for guarantee normal blood clotting and its level in newborns is commonly low, so a supplementation after delivery is mandatory.

Vitamin K prophylaxis in newborns is still an open field of debate: many types of protocol have been proposed in different years and Countries, and sometimes with great variability inside the same Nation (for instance, in Italy a national consensus is not available, so different protocols are employed). Recommendations include different protocols for healthy newborns born at term, but the unpreventable presence of bleeding favouring factors (i.e. blood vessels malformations) or limiting intestinal absorption of liposoluble vitamins (i.e. cholestasis), which could be unrecognized or subclinical in the perinatal period, rises some concerning about the most precautionary route of administration and the timing of further doses after the first one given at birth.

The purpose of this report is to underline the most recent evidences available in literature and to arise a debate about this topic, in order to stimulate the production of evidence-based guidelines concerning the prophylaxis with vitamin K1 in newborn infants, considering that many bleeding risk factors are not recognizable at birth.

**Case presentation:**

We are hereby presenting an emblematic case concerning the risk of intracranial bleeding in an apparently healthy newborn: the described infant did not show any pathological elements in pregnancy history or perinatal life which suggest a possible increased risk of bleeding and the needing of a particular approach in the administration of vitamin K1, but at the end of the first week of life presented an intracranial bleeding with neurological symptoms that required treatment for vitamin K deficiency.

**Conclusions:**

Univocal recommendations about vitamin K prophylaxis are not available and the contrast between oral and intramuscular routes persists unsolved. The difficulty to certainly identify an infant eligible for oral administration of vitamin K1 at birth suggests that the intramuscular route should be preferred. How to prosecute the supplementation in the first months of life is still an open topic of debate.

## Background

Preventing Vitamin K Deficiency Bleeding (VKDB) in newborns is an open challenge.

Vitamin K is a necessary cofactor of the synthesis of some coagulation factors (II, VII, IX and X) and anticoagulant proteins (C and S). The lack of vitamin K causes the production of undercarboxylated factors, unable to bind calcium and so to be activated [1].

We know that according to timing three types of Vitamin K deficiency bleeding are recognizable: early (within 24 h of life), classical (in the first week) and late (after the first week and before the sixth month). The first one is mainly related to employment in the mother of drugs (i.e. some antibiotics, drugs employed in epilepsy, anticoagulants) that interfere with vitamin K metabolism and could not be prevented through the administration of vitamin K to the newborn, but only giving it to the mother in the days before the delivery. The classical type is preventable through the administration of 1.0 mg of vitamin K to the newborn: oral or intramuscular route are both reported to be equally effective. The late type is the more dangerous and usually appear with intracranial bleeding: this form could not be completely prevented only through the administration of a single intramuscular dose of vitamin K [2, 3].

Hereby we are reporting an emblematic case concerning the risk of intracranial bleeding in an apparently healthy newborn.

## Case presentation

The infant was born at term through vacuum assisted delivery, without complications.

Pregnancy was uneventful, prenatal ultrasound evaluation did not report any pathological findings and serological screening for infectious diseases was negative. The mother did not assume any drugs that could interfere with vitamin K metabolism or blood clotting. Familiar pathological history did not report the recurrence of blood clotting abnormalities or early vascular accidents.

The infant presented good clinical conditions (APGAR score 9/9 at 1 and 5 min of life) and birth measures (weight, length and head circumference) were in non-pathological ranges. She got 2 mg of vitamin K1 through oral route at birth and parents received the indication to administer 50 mcg of vitamin K1 daily from the seventh day of life to the third month, according to the Italian Society of Neonatology protocol for healthy newborns born at term available at that time.

She underwent phototherapy for slight jaundice (bilirubin 13.3 mg/dL) during the fourth day of life and subsequently discharged from the hospital. She was exclusively breastfed.

**Fig. 1:**
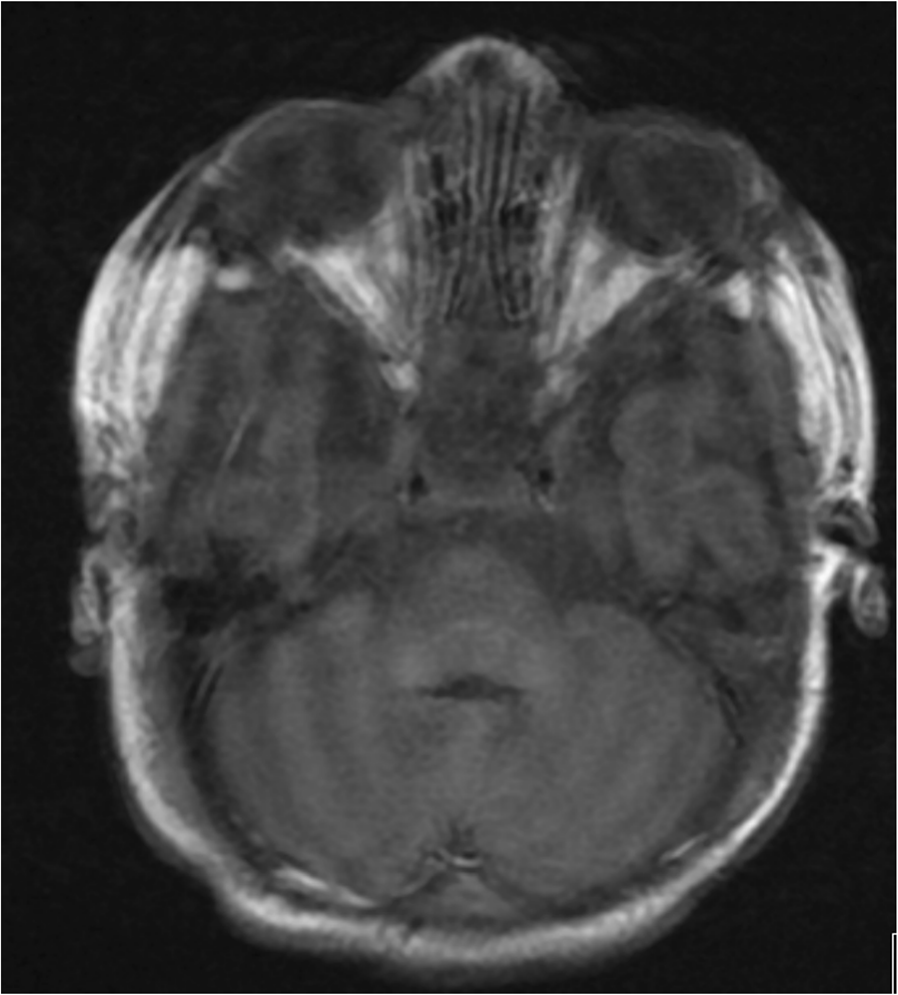
MRI T1 axial scan showing a bilateral bleeding along the cerebellar convexity

On the seventh day of life she presented epileptic episodes. First analysis revealed a slight increased PT (prothrombin time) ratio (1.26) and an increased aPTT (activated partial thromboplastin time) ratio (1.60). Platelet count was 286,000/mmc. In addition, a deficit of vitamin K dependent Protein C (17%) was detected. No mutations of Factor V Leiden and Prothrombin have been detected. Antiepileptic therapy with phenobarbital and vitamin B6 was started. Brain echography performed the same day did not show any pathological finding, while the one performed the following day showed parieto-occipital echogenic lesions, without clear signs of haemorrhage. So, cerebral MRI was performed [Fig. 1] and revealed a bilateral slight bleeding along the cerebellar convexity and also small haemorrhagic lesions have been detected in subcortical left frontal and right parietal regions. Taking into account haematological laboratory tests, time of onset of clinical signs and radiological evaluations, a classical type of VKDB was diagnosed, according to ESPGHAN definition [2]. Vitamin K1 (1 mg) was administered and treatment with vitamin K1 through intramuscular route 1 mg/kg/week was begun, with a progressive improvement of coagulation parameters and after two administrations PT ratio and aPTT ratio were normalized (respectively, 0.92 and 1.23). Vitamin K dependent clotting factors, after the first administration, resulted as follow: II (55%), VII (88%), IX (33%) and X (44%). Factors IX and X showed a progressive normalization.

Lab analysis also showed an increased value of gamma-glutamyl transpeptidase, that could be due to a subclinical cholestasis or be a consequence of the assumption of phenobarbital; however further investigations did not show a clear cholestatic liver disease.

## Discussion

The presented case is emblematic: an apparently healthy newborn infant, with no pathological findings in the pregnancy history, who suffered bleeding related to vitamin K deficiency and revealed unknown risk factors. She received vitamin K through the oral route at birth because she did not show any risk factors, but suffered a vitamin K deficiency bleeding (VKDB) at the end of the first week of life.

Intracranial haemorrhage in term infants are quite rare, involving from 1.6 to 5.9/10000 live births. These bleeding could be caused by coagulation disorders, perinatal hypoxic-ischemic events and traumatic mechanisms such as birth injury. Bleeding could be more frequently intraventricular (usually diagnosed during fetal life through ultrasonography) or intraparenchymal, whereas subarachnoid and subdural haemorrhages are rarer [4].

Up to now, plenty protocols of prophylaxis with vitamin K could be find: differences are detectable from a Country to another and sometimes between different hospitals belonging to the same Nation (for instance, in Italy is not available a national recommendation). However, Countries which recommended intramuscular administration usually show a lower incidence of bleeding due to vitamin K deficiency [1, 5].

Oral administration of vitamin K could be inefficient because of cholestasis (1 in 2500 live births) [3, 6], especially in unrecognized or subclinical cases. In children affected by this disease vitamin K deficiency could last longer and, because of the lack of intestinal absorption, only further administration (2–5 mg/monthly) of intramuscular vitamin K or higher doses (2.5–5 mg two times/week) given orally could prevent bleeding [6]. Infants affected by malabsorption or cholestasis are not protected from vitamin K deficiency if they receive daily low doses of vitamin K1 (25 mcg) [7, 8].

Oral administration at birth has been compared to intramuscular route in a population of children affected by biliary atresia and the second way is demonstrated to be the most efficient in preventing classical and late types of VKDB [9].

In addition, oral route is affected by other variabilities: for instance, absorption of oral drops given in isolation is lower than the one of the same dosages contained in a formula [3] and blood levels of vitamin K1 are lower in breastfed infants than in formula fed ones [2].

A survey conducted in Japan by Takahashi et al. [10] reports that in 63 (88.7%) observed cases of late VKDB, vitamin K had been administered orally at least once during the first month of life, so we could speculate that the oral route could be not effective in preventing late episodes; alternatively, higher dosages or more administrations may be required. So, the prevention of late VKDB is clearly another hot topic of debate, as underlined by Unal et al. and Pirinccioglu et al. in their Papers [11, 12], in which they underline how current protocols sometimes need to be modified and that a single dose of vitamin K is not sufficient to prevent late VKDB (they analyzed the Turkish situation).

ESPGHAN Committee on Nutrition has recognized that the intramuscular application is the favourite way for efficiency and reliability, however stated that oral administration could be available for healthy not-preterm newborn infants. According to its position paper, this population of infants could receive vitamin K as follow: 1 mg intramuscular at birth or 2 mg orally at birth, at 4 to 6 days and at 4 to 6 weeks or 2 mg orally at birth, followed by a weekly dose of 1 mg for 3 months [2]. The Committee also underlined that oral route could be affected by a great variability of absorption (i.e. compliance, setting, unknown cholestasis, presence of vomiting or regurgitation) [2].

## Conclusions

Univocal recommendations about vitamin K prophylaxis are not available. We could be inspired by the guidelines of other European countries, but anyway the contrast between oral and intramuscular routes persists unsolved, as reflected by the statement of Espghan.

Oral way may be suitable for healthy not-preterm newborn infants, but we must remember that many pathological conditions affecting the absorption of liposoluble vitamins or favouring bleeding are not clearly recognizable at birth. This issue requires a careful approach: should we perform a more depth screening at birth? Or should we begin a stricter clinical follow up in the postnatal age, maybe considering the possibility of vitamin K1 blood dosage or the dosage of some related factors?

At birth, clinical tools to certainly identify an infant eligible for oral administration are not available, so intramuscular administration route seems to be more reliable.

How to prosecute the supplementation of vitamin K1 in the first months of life is still an open topic of discussion: in Italy, last recommendations of the Italian Society of Neonatology suggest to prosecute the administration orally with 150 mcg daily until the fourteenth week if the first dose at birth was given by mouth in not-preterm infants. However, we have to consider that in malabsorption a low daily oral dosage may not be useful in preventing deficit as described in the discussion section, so other protocols of oral supplementation in the first months of life should be considered.

## Abbreviation

VDKB: vitamin K deficiency bleeding
